# Associations between fatigue and physical capacity in people moderately affected by rheumatoid arthritis

**DOI:** 10.1007/s00296-018-4140-z

**Published:** 2018-08-29

**Authors:** Ingrid Demmelmaier, Susanne Pettersson, Birgitta Nordgren, Alyssa B. Dufour, Christina H. Opava

**Affiliations:** 1 Division of Physiotherapy, Department of Neurobiology, Care Sciences and Society, Karolinska Institutet, Stockholm, Sweden; 2 Theme Inflammation and Infection, Karolinska University Hospital, 141 86 Stockholm, Sweden; 3 Functional Area Occupational Therapy and Physiotherapy, Allied Health Professionals’ Function, Karolinska University Hospital, Stockholm, Sweden; 4 Harvard Medical School, Institute for Aging Research, Hebrew Senior Life, Boston, MA USA; 5 Beth Israel Deaconess Medical Center, Boston, MA USA; 6 Rheumatology Clinic, Karolinska University Hospital, Stockholm, Sweden

**Keywords:** Aerobic capacity, Fatigue, Grip strength, Lower limb function, Physical capacity, Rheumatoid arthritis

## Abstract

To explore the contribution of physical capacity in explaining variations in fatigue among people with rheumatoid arthritis (RA). This study included participants recruited for a physical activity intervention. Data were collected from the Swedish Rheumatology Quality Registers, from questionnaires on fatigue, activity limitation, perceived health, pain and anxiety/depression and from physical capacity tests (lower limb function, grip strength, and aerobic capacity). We used logistic regression to estimate the association between severe fatigue (≥ 50, visual analogue scale 0–100) and (A) independent variables related to disease and disease impact and (B) model A plus physical capacity tests. Pooled odds ratio tests compared model fit. Out of the 269 participants (mean age 60 years, mean disease activity score [DAS28] 2.8), severe fatigue was reported by 35%. The three variables which were statistically significantly associated with severe fatigue (*p* < 0.05) in both models were perceived health, pain and anxiety/depression. Anxiety/depression demonstrated the largest effect size with odds ratios of 2.43 (95% CI 1.20, 4.94) in model A and 2.58 (95% CI 1.25, 5.32) in model B. The likelihood ratio test indicated that model B was a better fit to the data than model A with *Χ*^2^ (*df* 3) = 2.65, *p* = 0.048. Severe fatigue in people with RA is associated with self-rated health, pain and anxiety/depression rather than with physical capacity. Future studies should be prospective, use multidimensional assessments of fatigue to explore the influence of physical capacity and control for possible influence of comorbidities associated with fatigue.

## Introduction

Rheumatoid arthritis (RA) is an inflammatory disease and its impact on patients is traditionally categorized in high, moderate, low or remission as captured by a disease activity score [1]. Disease impact reflected as activity limitation can be stratified based on the Health Assessment Questionnaire (HAQ) [2]. Additional aspects of RA impact can be captured as general health perception or quality of life [3]. With modern medical treatment disease impact seems to be less prominent [4], but several disease aspects, e.g., fatigue and pain, still impair patients’ daily life despite low-disease activity [5].

Fatigue is one of the most prominent problems in people with rheumatic diseases and has often a detrimental effect on their quality of life [6]. Fatigue may be peripheral or central [7] with the former deriving from neuromuscular dysfunction related to impaired neurotransmission in the peripheral nerves and/or deficits in muscular contraction, and the latter described as abnormalities in neurotransmitter pathways within the central nervous system (CNS). Central fatigue is presumably more frequent in people with chronic conditions and more influenced by psychological complaints.

A theoretical model of fatigue in RA suggests that several interrelated factors contribute to the experience and burden of fatigue [8]. The model includes disease-related factors, such as pain or inflammation, as drivers of fatigue. However, the most evident correlates of fatigue are pain, physical impairment and depression [9], with depression suggested as a mediator influenced by disease activity [10]. Furthermore, pain and fatigue often mutually influence and interfere with each other [11]. Hence, pain may also explain, to some extent, conflicting reports on correlations between fatigue and disease activity in RA [12].

Physical activity, including planned and structured exercise, is an important non-pharmacological intervention that positively affects fatigue in RA [13, 14]. Additionally, physical activity also positively influences disease activity [15], aerobic capacity and muscle strength [16], pain, activity limitation and health-related quality of life [16]. Furthermore, qualitative aspects of physical activity have been described in terms of joy, wellbeing and satisfaction, identification with wellness rather than illness and expanding social networks [17]. Thus, the influence of physical activity on fatigue might be a mix of biological, physical and psychosocial benefits. To our knowledge, only one previous study has explored the relative impact of a comprehensive set of such determinants on fatigue in RA. Aerobic capacity was the only physical capacity test included and did not explain the variance in fatigue, which was mainly explained by depression [18]. Not only aerobic capacity but also muscle strength and lower limb capacity, are modifiable and their relative contributions to fatigue are thus of interest to better understand and address this detrimental consequence of RA.

The aim of the present study was to explore the contribution of physical capacity (aerobic capacity, grip strength and timed standing) in explaining variations of fatigue among people with RA in addition to predictors previously identified.

## Materials and methods

### Design and participants

This cross-sectional study used baseline assessment data from 269 people diagnosed with RA according to the American Rheumatism Association criteria [19] who had agreed to participate in a 2-year support program for health-enhancing physical activity (HEPA) [20]. The study sample, for which the selection procedure has previously been described in detail [21], was derived from a larger sample identified from six rheumatology clinics via the Swedish Rheumatology Quality Registers (SRQ) [22]. The participants of the present sample were 18–75 years of age, independent in daily living (Stanford Health Assessment Questionnaire disability index, HAQ-DI ≤ 2) [23], not physically active in line with HEPA guidelines [24], had good Swedish language skills and indicated interest to participate in a HEPA trial [21]. No additional exclusion criteria were applied.

### Assessment methods

#### Dependent variable

Fatigue was rated on a 100 mm visual analogue scale (VAS) from 0 (“No fatigue”) to 100 (“Maximal fatigue”). The fatigue VAS has good face validity and is sensitive for change in RA [25].

#### Independent variables

*Sociodemographics* Age and gender were retrieved from the SRQ, education (university or not) and living single or with others were self-reported in a questionnaire specifically designed for the present study.

*Disease-related variables* Disease duration (years since first symptoms), disease activity 28-joint count (DAS28) calculated on erythrocyte sedimentation rate (ESR), medication with disease-modifying antirheumatic drugs (DMARDs) (yes/no) and medication with biologics (yes/no) were retrieved from the SRQ and/or patient files. Comorbidities were defined as report of any of the categories “respiratory”, “cardiovascular”, “neurological” “psychiatric” disease, “diabetes mellitus” or “other”.

*Self-reported physical activity and anthropometrics* Current HEPA (past week) was assessed by the International Physical Activity Questionnaire (IPAQ)—short version [26] and described as obtained or not, based on the IPAQ scoring protocol [27]. Maintained HEPA (past 6 months) was assessed by the exercise stage assessment instrument (ESAI) [28] and described as obtained or not. Body mass index (BMI) was calculated as body weight (kg) divided by the square of body height (m^2^).

*Perceived disease impact* Activity limitation was assessed by the Stanford Health Assessment Questionnaire-Disability Index (HAQ-DI) and ranged from 0 (“no activity limitation”) to 3 (“totally dependent”) [23]. Perceived health was rated on a 100 mm visual analogue scale (VAS) from 0 (“totally fine”) to 100 (“worst imaginable health”) and pain with VAS from 0 (“no pain”) to 100 (“maximal pain”) [29]. Anxiety/depression was assessed with one item from the EuroQol (EQ-5D-3L) ranging from 1 to 3, with 1 indicating “no problems” and 3 “extreme problems” [30]. The EuroQol is not a diagnostic tool, but rather captures the perception of distress.

*Physical capacity* Lower limb function was assessed with the timed-stands test (TST) and presented in seconds (s) [31] and average grip strength was assessed with the Grippit device (N) [32]. The mean of three 10-s grip force trials of the right hand was used for analyses. The results of lower limb function and grip strength tests were categorized into normal or below normal reference values [31, 32]. Maximal oxygen uptake (ml × kg^1^ × min^1^) was estimated from a submaximal Åstrand and Rhyming bicycle ergometer test based on a linear relationship between mechanical load, oxygen uptake and heart rate obtained during the test [33].

### Data management and statistical analyses

The dependent variable fatigue was categorized as low/moderate (0–49 mm) or severe (50–100 mm) as previously used in RA [12]. Descriptive statistics (means, standard deviations or proportions) were calculated for the total sample and stratified by level of fatigue. Multiple imputation (package MICE in R) was used to impute missing data among the independent variables [34]. Pooled odds ratios (OR) and 95% confidence intervals (CI) for the univariate association between each independent variable and severe fatigue were calculated using pooled logistic regression (package MICE in R). Variables significantly associated with severe fatigue in univariate analyses (*p* < 0.1) were included in two adjusted logistic regression models: (A) including variables related to disease and disease impact (disease duration, biological drugs, comorbidities, activity limitation (HAQ-DI), perceived health, pain, anxiety/depression) and (B) including variables related to disease, disease impact and physical capacity (grip strength, lower limb function, aerobic capacity). Pooled odds ratios and 95% confidence intervals were calculated using pooled logistic regression (package MICE in R) and models A and B were compared using the pooled likelihood ratio test for multiple imputations [35]. An alpha level of 0.05 was used in the adjusted logistic regression models to indicate statistically significant results.

### Ethics

The study was carried out in compliance with the Helsinki Declaration. Ethics approval was obtained from the Stockholm Regional Ethical Review Board. Participation was sought in a letter containing information about the study, and the participants consented by returning their questionnaires.

## Results

The 269 participants had predominantly low/moderate disease activity and moderate impact of the disease (Table 1). A majority of the participants had normal lower limb function (76%) and normal grip strength (74%) according to age and gender match norm values. Participants’ mean level of fatigue was 36 (SD 26) and 35% (n = 95) were categorized as having severe fatigue (≥ 50).

**Table 1:** Characteristics of the total sample and the subgroups with low/moderate fatigue (VAS 0–49) and severe fatigue (VAS 50–100)

	Total sample (*n* = 269)	Subgroup with low/moderate fatigue (*n* = 174)	Subgroup with severe fatigue (*n* = 95)
*Sociodemographics*			
Age years, mean (SD)	60 (9)	60 (9)	59 (9)
Gender			
Female, *n* (%)	220 (82)	139 (80)	81 (85)
Male, *n* (%)	49 (18)	35 (20)	14 (15)
Education			
No university, *n* (%)	136 (51)	87 (50)	49 (52)
University, *n* (%)	133 (49)	87 (50)	46 (48)
Other adults in household			
No, *n* (%)	62 (23)	38 (22)	24 (25)
Yes, *n* (%)	205 (76)	135 (78)	70 (74)
*Disease-related variables*			
Disease duration yrs, mean (SD)	12 (9)	11 (9)	14 (11)
Disease activity DAS28 0–10, mean (SD)	2.8 (1.2)	2.8 (1.1)	3.1 (1.3)
Remission 0–2.5, *n* (%)	89 (33)	65 (37)	24 (25)
Low 2.6–3.1, *n* (%)	38 (14)	22 (13)	16 (17)
Moderate 3.2–5.1, *n* (%)	61 (23)	38 (22)	23 (24)
High 5.2–10, *n* (%)	12 (4)	7 (4)	5 (5)
Medication DMARDs			
No, *n* (%)	49 (18)	33 (19)	16 (17)
Yes, *n* (%)	163 (61)	106 (61)	57 (60)
Medication biological drugs			
No, *n* (%)	94 (35)	67 (39)	27 (28)
Yes, *n* (%)	123 (46)	75 (43)	48 (51)
Comorbidities			
No, *n* (%)	116 (43)	82 (47)	34 (36)
Yes, *n* (%)	153 (57)	92 (53)	61 (64)
*Perceived disease impact*			
Activity limitation HAQ-DI 0–3, mean (SD)	0.5 (0.5)	0.4 (0.4)	0.8 (0.5)
No, (0), *n* (%)	63 (23)	57 (33)	6 (6)
Yes, (> 0) *n* (%)	206 (77)	117 (67)	89 (94)
Health VAS 0-100, mean (SD)	30 (21)	20 (13)	49 (19)
Pain VAS 0-100, mean (SD)	28 (22)	18 (14)	47 (21)
Fatigue VAS 0 -100, mean (SD)	36 (26)	20 (15)	66 (12)
Anxiety/depression			
EQ5D-3L 1–3, mean (SD)	1.3 (0.5)	1.2 (0.4)	1.6 (0.6)
*Self-reported physical activity*			
Current HEPA IPAQ			
No, *n* (%)	109 (41)	65 (37)	44 (46)
Yes, *n* (%)	160 (59)	109 (62)	51 (54)
Maintained HEPA ESAI			
No, *n* (%)	228 (85)	148 (85)	80 (84)
Yes, *n* (%)	38 (14)	23 (13)	15 (16)
*Physical capacity and anthropometrics*			
Lower limb function TST s, mean (SD)	22 (9)	22 (7)	23 (12)
Low, *n* (%)	57 (21)	36 (21)	21 (22)
Normal, *n* (%)	205 (76)	134 (77)	71 (75)
Grip strength Grippit (*N*), mean (SD)	194 (114)	204 (110)	175 (120)
Low, *n* (%)	69 (26)	32 (18)	37 (39)
Normal, *n* (%)	198 (74)	141 (81)	57 (60)
Aerobic capacity Åstrand bicycle test, ml × kg^1^ × min^1^, mean (SD)	28 (8)	28 (8)	28 (9)
Body mass index kg/m^2^, mean (SD)	27 (5)	26 (5)	27 (5)

For perceived disease impact variables, high values indicate high impact

When percentages within variables do not add up to 100, there are missing values

*DAS28* Disease Activity Score 28, *DMARDs* disease-modifying antirheumatic drugs, *HAQ-DI* Stanford Health Assessment Questionnaire, *VAS* Visual Analogue Scale, *EQ5D-3L* euro quality of life 5 dimensions, *HEPA* health-enhancing physical activity, *IPAQ* International Physical Activity Questionnaire, *ESAI* exercise stage assessment instrument, *TST* timed-stands test

In the univariate analyses, significant associations (*p* < 0.1) between severe fatigue and disease duration, medication with biological drugs, presence of comorbidities, activity limitation, perceived health, pain, anxiety/depression and grip strength were found (Table 2).

**Table 2:** Univariate associations between potential predictors and being in the severe fatigue category

	OR (95% CI)	*p* value
*Sociodemographics*		
Age, years	0.99 (0.96, 1.02)	0.446
Gender, male vs female	0.69 (0.35, 1.35)	0.277
Education university vs not university	0.94 (0.57, 1.55)	0.804
Other adults in household^a^, yes vs no	0.84 (0.46, 1.51)	0.554
*Disease-related variables*		
Disease duration, years	1.02 (1.00, 1.05)	0.080
Disease activity^a^ 0–10	1.18 (0.93, 1.51)	0.176
Medication DMARDs^a^, yes vs no	1.13 (0.56, 2.28)	0.740
Medication biological drugs^a^, yes vs no	1.67 (0.93, 3.00)	0.086
Comorbidities, yes vs no	1.60 (0.96, 2.67)	0.074
*Perceived disease impact*		
Activity limitation 0–3	5.40 (3.05, 9.54)	< 0.0001
Health^a^ 0–100	1.11 (1.08, 1.13)	< 0.0001
Pain 0–100	1.08 (1.06, 1.10)	< 0.0001
Anxiety/depression1–3	4.23 (2.50, 7.16)	< 0.0001
*Self-reported physical activity*		
Current HEPA, yes vs no	0.69 (0.42, 1.15)	0.153
Maintained HEPA^a^, yes vs no	1.17 (0.58, 2.37)	0.658
*Physical capacity and anthropometrics*		
Lower limb function^a^ seconds	1.02 (0.99, 1.05)	0.159
Grip strength^a^ Newton	1.00 (1.00, 1.00)	0.053
Aerobic capacity^a^ ml × kg^1^ × min^1^	0.99 (0.96, 1.03)	0.653
Body mass index^a^ kg/m^2^	1.02 (0.97, 1.08)	0.389

Odds ratios (OR) and 95% confidence intervals (CI) for 1 unit increase in each predictor

Abbreviations: see Table 1

^a^Multiple imputation for missing data

Results from the two adjusted logistic regression models are presented in Table 3. The three variables which were statistically significantly associated with severe fatigue (*p* < 0.05) in both models were perceived health, pain and anxiety/depression. Anxiety/depression demonstrated the largest effect size with odds ratios of 2.43 (95% CI 1.20, 4.94) in model A and 2.58 (95% CI 1.25, 5.32) in model B. The likelihood ratio test indicated that model B was a better fit to the data than model A with *Χ*^2^ (*df* 3) = 2.65, *p* = 0.048.

**Table 3:** Outcome of logistic regression for severe fatigue with odds ratios (OR) and 95% confidence intervals (CI) for 1 SD increase in each predictor variable

Model A	OR (95% CI)	*p* value	Model B	OR (95% CI)	*p* value
Disease duration^a^	0.97 (0.93, 1.01)	0.169	Disease duration^a^	0.97 (0.93, 1.02)	0.277
Biological drugs^b^	0.84 (0.37, 1.92)	0.679	Biological drugs^b^	0.86 (0.36, 2.08)	0.742
Comorbidities^c^	1.73 (0.81, 3.71)	0.160	Comorbidities^c^	2.05 (0.92, 4.59)	0.079
Activity limitation^d^	1.29 (0.53, 3.17)	0.571	Activity limitation^d^	2.24 (0.83, 6.1)	0.113
Health^e^	1.07 (1.04, 1.11)	< 0.0001	Health^e^	1.08 (1.04, 1.12)	< 0.0001
Pain^f^	1.04 (1.02, 1.07)	0.002	Pain^f^	1.04 (1.01, 1.07)	0.004
Anxiety/depression^g^	2.43 (1.2, 4.94)	0.014	Anxiety/depression^g^	2.58 (1.25, 5.32)	0.011
			Grip strength^h^	1 (1, 1.01)	0.167
			Lower limb function^i^	0.95 (0.91, 1)	0.051
			Aerobic capacity^j^	1.02 (0.96, 1.08)	0.608

Results produced using R, package mice [33]

Model A includes disease-related and perceived disease impact variables and Model B also includes physical capacity variables (*n* = 269)

Pooled likelihood ratio test to compare models = 2.65, *p* = 0.048

^a^Disease duration, years

^b^Medication with biological drug, yes vs no

^c^Comorbidities, yes vs no

^d^Activity limitation, 0–3

^e^Perceived health, 0–100

^f^Pain, 0–100

^g^Anxiety/depression, 1–3

^h^Grip strength, N

^i^Lower limb function, s

^j^Aerobic capacity, ml × kg^1^ × min^1^

## Discussion

To our knowledge, this is the first study investigating the explanation of fatigue by physical capacity, other than aerobic capacity. However, in contrast to our assumptions, we only found physical capacity to be marginally associated with severe fatigue in this sample moderately affected with RA. Its contribution was only indicated by better model fit by inclusion of physical capacity variables, whereas the previously known disease impact variables perceived health, pain and anxiety/depression consistently and significantly contributed to variations in fatigue.

The participants in the present study were moderately affected by RA, with more than half of them in remission or with low-disease activity. Additionally, a majority of our sample had normal lower limb function [31] and normal grip strength [36] and only 35% of them reported severe levels of fatigue, which is comparably low [25]. Despite this, our results confirm previous findings demonstrating perceived health and pain as important contributors [9, 37] and previous results from studies with limited sample sizes suggesting that aerobic capacity does not contribute to explain self-reported levels of fatigue [18, 38]. Our findings of very limited contribution of physical capacity in explaining severe fatigue might partly be explained by our moderately affected sample. Expected correlation between self-reported variables and measured physical capacity is another likely explanation for our results.

Relations between physical activity, fatigue and disease activity in people with RA are complex. Additional factors such as sleep and pain contribute, directly or indirectly, to the burden of fatigue [39]. High levels of fatigue reduce participation in physical activity [40], while on the other hand physical activity has the potential to reduce fatigue [13, 14, 41]. Similarly, there is no evidence of physical activity increasing disease activity, which may rather be reduced following exercise [42]. However, disease severity with widespread joint involvement might still limit physical activity [40].

The strongest associations with fatigue were found for anxiety/depression in both models explored in the present study. This is consistent with previous studies demonstrating mood disorders to have both a direct and indirect effect on fatigue in persons with RA [10, 43]. Since physical activity is important in the non-pharmacological treatment of depression [44], our results reinforce the importance of incorporating PA in the treatment of both depression and fatigue in RA.

Increased oxygen consumption, and the consequence metabolic cost, has previously been suggested as one plausible link to fatigue in RA [45]. In our study, the absence of associations, between fatigue and physical capacity, might be explained by our relatively fit sample compared to other RA samples, and values close to those of healthy individuals [46]. However, this result is somewhat puzzling since none of the participants fulfilled criteria for maintained (≥ 6 months) levels of health-enhancing physical activity [24].

The major strengths of our study are the well-defined sample, our use of validated self-report questionnaires and capacity measures, and that the physical therapists conducting the capacity tests were trained to adhere to established test procedures. However, our assessment of fatigue with the use of a single-item measure, reflecting a global perspective of fatigue rather than a more multidimensional [47] may be questioned. On the other hand, the VAS was among the six questionnaires to assess fatigue recommended by the OMERACT 8 in 2006 when fatigue was included in the recommended core set for studies of persons with RA [48]. Another potential methodological bias might be our choice of a cut-off for severe fatigue that might be too low to result in convincing findings. However, the cut-off chosen is well documented and has been used in several previous studies [12, 49]. Furthermore, a higher cut-off for severe fatigue would have resulted in too small groups for statistically meaningful analyses in the present sample. Another possible limitation might be our use of self-reported physical activity that may be influenced by e.g., recall bias and social desirability. However, it is easy, affordable, and therefore, feasible for large groups of people. Objective recording of physical activity has become more feasible and affordable, but might still suffer from certain validity issues caused by limited wear time; periods during which participants, due to social desirability, might assume different physical activity patterns than their normal ones. Furthermore, possible selection bias cannot be excluded since overrepresentation of people interested in physical activity and underrepresentation of those with less interest, and maybe higher levels of fatigue. This potential threat to external validity should be considered when interpreting our results.

Fatigue in RA is complex and includes both biological and psychological aspects contributing to its burden on individuals [8, 39]. Additionally, potentially coexisting conditions such as chronic fatigue syndrome, low vitamin D level, osteoporosis and infectious diseases also include a panorama of symptoms comprising fatigue. Neither biological mechanisms behind fatigue in RA nor differences and similarities in origin of fatigue compared to other conditions are completely defined. While exploration of such coexisting reasons for fatigue were beyond the scope of our study, they should be included in future studies to contribute to a more complete understanding of fatigue.

Since our study was cross-sectional, future longitudinal studies with repeated measures of fatigue to explore causation and identify predictors of fatigue are needed [9]. Further, the use of a multidimensional assessment of fatigue has the potential to increase our understanding of the role of physical capacity in explaining components of fatigue, e.g., mental and physical, and should be considered for inclusion in future studies on explanation of fatigue. This should preferably be done in large samples and with more physical capacity variables. In future studies, including physical capacity, we also suggest that the calculation of estimated maximal oxygen uptake based on the submaximal Åstrand test [33] should be replaced by other algorithms that may be more accurate for the RA population [50].

## Conclusions

Our results indicate that severe fatigue in people with RA is associated with self-rated health, pain and anxiety/depression rather than with physical capacity. Future studies should be prospective and use multidimensional assessments of fatigue to explore the influence of physical capacity on physical and mental fatigue, respectively, and the possible influence of comorbidities associated with fatigue should be controlled for.
